# Sleeve-gastrectomy results in improved metabolism and a massive stress response of the liver proteome in a mouse model of metabolic dysfunction-associated steatohepatitis

**DOI:** 10.1016/j.heliyon.2024.e38678

**Published:** 2024-09-28

**Authors:** Andreas Kroh, Sophia Schmitz, Saskia Köhne, Julia Andruszkow, Jochen Nolting, Christian Preisinger, Karsten Große, Roman M. Eickhoff, Daniel Heise, Thorsten Cramer, Karl Peter Rheinwalt, Patrick Hamid Alizai, Ulf Peter Neumann, Tom Florian Ulmer

**Affiliations:** a General-, Visceral-, Pediatric and Transplant Surgery, RWTH Aachen University Hospital, Pauwelsstr. 30, 52074, Aachen, Germany; b General-, Visceral- and Transplant Surgery, Uniklinik Essen, Hufelandstr. 55, 45147, Essen, Germany; c Institute of Pathology, RWTH Aachen University Hospital, Pauwelsstr. 30, 52074, Aachen, Germany; d Proteomics Facility, Interdisciplinary Center for Clinical Research (IZKF) Aachen, RWTH Aachen University Hospital, Pauwelsstr. 30, 52074, Aachen, Germany; e Department of Medicine III, RWTH Aachen University Hospital, Pauwelsstr. 30, 52074 Aachen, Germany; f Department for Bariatric and Metabolic Surgery, St. Franziskus Hospital, Cologne, Germany; g Department of General and Visceral Surgery, Gemeinschaftskrankenhaus Bonn, Bonn, Germany

**Keywords:** Sleeve-gastrectomy, Metabolic dysfunction-associated steatohepatitis, Nonalcoholic steatohepatitis, Metabolic dysfunction-associated steatotic liver disease, Nonalcoholic fatty liver diseases, Bariatric surgery

## Abstract

**Background:**

Bariatric surgery has been shown to improve the histopathological findings in patients with obesity and metabolic dysfunction-associated steatohepatitis, but there are also reports about non-responders or progressive disease after bariatric interventions. Therefore, it is of utmost importance to understand the pathophysiological processes in the liver after bariatric surgery.

**Materials and methods:**

In the present study, 4 weeks old male C57/Bl6 mice were fed a Western Diet to induce metabolic dysfunction-associated steatohepatitis and sleeve-gastrectomy (SG), or sham operation in the pair-fed and ad libitum control group were performed. Mice were observed for two or eight weeks after surgery and metabolic assessment was performed throughout the experiment. Histopathology, flow cytometry and proteomic analyses were conducted to evaluate hepatic inflammation, liver metabolism and affected signaling pathways.

**Results:**

Weight loss was higher, and metabolism significantly improved after SG. Two weeks after SG major inflammatory and regulatory disturbances in the liver were observed. The proportion of hepatic CD3^+^NK1.1^+^ cells were decreased, and proteins involved in apoptosis like Fas, Casp1 and Casp9 or in the acute phase response were upregulated in SG mice. These disturbances decreased in the long-term and we observed an increase of many proteins involved in lipid metabolism eight weeks following SG.

**Conclusions:**

The rapid weight loss and decrease of hepatic fat after SG lead to a proinflammatory response in the liver in the early phase after surgery, which changes to a more moderate immune response in the long-term. We suggest a preoperative risk stratification and postoperative surveillance depending on the histopathological findings.

## Highlights

• Inflammatory disturbances and a stress response in the liver are prominent in the initial course after sleeve-gastrectomy.
• Over the long term, sleeve-gastrectomy results in substantial weight loss and improvement of both metabolism and liver pathophysiology.
• Preoperative risk assessment is essential for counseling patients effectively and delivering individualized therapeutic strategies.

## 1 Introduction

Metabolic dysfunction-associated steatotic liver disease (MASLD) and metabolic dysfunction-associated steatohepatitis (MASH), as an inflammatory manifestation of MASLD, are the most common chronic liver diseases today and occur as part of the metabolic syndrome in obese patients. In Western industrialized countries, up to 46 % of the population shows signs of MASLD and up to 12.2 % suffer from manifest MASH [1,2]. The exact mechanism behind the development of MASH has not been finally elucidated to date, but obesity and insulin resistance are verified risk factors [[3], [4], [5], [6]]. Adipose tissue itself, as an endocrine active organ, is also involved in chronic inflammatory processes and the pathogenesis of MASH through altered secretion of pro- and anti-inflammatory factors [7]. Moreover, chronic inflammation is maintained by bacterial components from the enterohepatic circulation [8]. Unfortunately, the treatment of obesity itself and of obesity-associated MASH is difficult. In addition to the long-term failure of lifestyle modifications (exercise, dieting) [9], even new drug therapies suffer from side effects and depend on long-term therapy [10,11].

Bariatric surgery is currently the only therapeutic option for morbid obesity whose efficacy has been demonstrated in extensive meta-analyses [12,13]. Importantly, not only weight is reduced, but obesity-associated diseases including MASH are also effectively treated [14]. In a study, in patients with histologically confirmed MASH, 85 % of the patients showed complete resolution of MASH one year after the bariatric intervention [15]. Unfortunately, the underlying mechanisms remain elusive; however, several hepatocellular injury signals associated with MASLD and MASH, such as lipotoxicity and endoplasmic reticulum stress, are alleviated following bariatric procedures. Altered gastrointestinal hormones, such as ghrelin, may also contribute to these effects [16,17].

On the other hand, there have also been reports about cases of acute liver failure or even liver cirrhosis following bariatric procedures [18,19]. However, it is certain that the metabolic and inflammatory disturbances after bariatric surgery in MASH patients are not fully elucidated.

Therefore, the aim of this study is to evaluate the impact of sleeve-gastrectomy on the pathogenesis of MASH. Here, the endocrine and inflammatory changes that occur in the context of obesity will be correlated with liver inflammation with a particular focus on changes of the liver proteome.

## 2 Materials and methods

### 2.1 Animals

All animal experiments were conducted with the approval of the governmental animal care and use committee (LANUV) in Recklinghausen, North Rhine-Westphalia, Germany. The study received official authorization (84-02.04.2014.A356) and was carried out in accordance with German federal law and European Directive 2010/63/EU on the protection of animals used for scientific research. Additionally, our procedures adhered to the ARRIVE guidelines and followed the recommendations of the “Guide for the Care and Use of Laboratory Animals” (8th edition, NIH publication, 2011, USA). Male C57/Bl6J mice, obtained from Charles River Laboratories (Germany), were used in the experiments. Female mice were excluded to avoid sex differences in obesity-related complications and metabolic syndrome. The animals were housed in specific pathogen-free (SPF) conditions according to FELASA guidelines (www.felasa.org), maintained on a 12-h light/dark cycle, and acclimated for two weeks on a standard chow diet (SC, V1534-300, sniff GmbH, Germany) at the RWTH Aachen animal facility before starting the experiments. The animals were monitored daily, with their clinical condition evaluated weekly by an experienced technician using a scoring system adapted from Kanzler et al. for animal welfare assessment [20]. (Table 1, Table 2).

**Table 1:** Clinical scoring system for animal welfare assessment.

Parameter		Score
**General state**		
Body weight∗	Variation <5 %	1
	Weight reduction 5–10 %	5
	Weight reduction 11–20 %	10
	Weight reduction >20 %	20
Fur	Fur faulty (reduced body hygiene)	1
	Fur lusterless and disheveled	5
	Fur dirty	10
Stoma	Scruffy	1
	Sticky or moist	5
Eyes	Turbid	5
**Behavior**		
Nutrition	Hypophagia	5
	Fasting	10
Activity	Limited motor function or hyperkinetic	5
	Ducked posture, lethargy, coordination disorder	10
Social behavior	Self-isolation	5
	Missing fight-or-flight response	10
	Self-amputation (automutilation)	20
**Clinical results**		
Digestion	Diarrhea	5
	Steatorrhea	5
Pulse and respiration	Respiration and pulse ±30 %	5
	Respiration and pulse ±50 %	20
Vegetative symptoms	Shivering	10
Trial-specific indicators	Impaired wound healing	5
	Anastomotic leakage	20
	Bleeding	20
	Edema, ascites or jaundice	20

**Table 2:** Degree of strain (DS) and procedures.

DS	Category	Procedure	Score
DS0	No strain		0
DS1	Minor category	careful observation necessary	1–4
DS2	Moderate category	supportive measures (additional analgesia or fluids)	5–9
DS3	Critical category	DS2 + veterinary support	10–19
DS4	High-grade strain	consult animal protection commissary, veterinary support necessary, termination of experiment, euthanasia	≥20

### 2.2 Study design

In this study mice were divided into three groups: a Sleeve-Gastrectomy (SG), and two sham operation groups. Sham operation groups included an ad libitum fed control and a Pair-fed (Pf) group. Pair-fed animals only received that amount of food SG mice had eaten at the same postoperative timepoint during a 24-h time-period. To examine short and long-time effects of SG, mice were either observed for two or eight postoperative weeks. Starting at four weeks of age, the mice were provided with water enriched with 10 % sucrose and fed a Western diet, consisting of 40 % of calories from fat, 20 % from fructose, and 2 % from cholesterol (Western diet; WD; cat# D09100301, Research Diets, Inc., New Brunswick, NJ, USA), to induce metabolic syndrome. Their weight and food intake were recorded weekly. After 12 weeks on this diet, the mice in each cage were randomly allocated to either a sleeve gastrectomy (SG) or sham surgery group (Fig. 1c). Blood tests and intraperitoneal glucose tolerance tests (IP-GTT) were performed at baseline (before staring the diet), five days before surgery, two weeks after surgery, and prior to the planned sacrifice. After the mice were euthanized, the livers were excised, divided and snap frozen in liquid nitrogen or fixed in 4 % paraformaldehyde. The study design is depicted as timeline in Fig. 1.

**Fig. 1:**
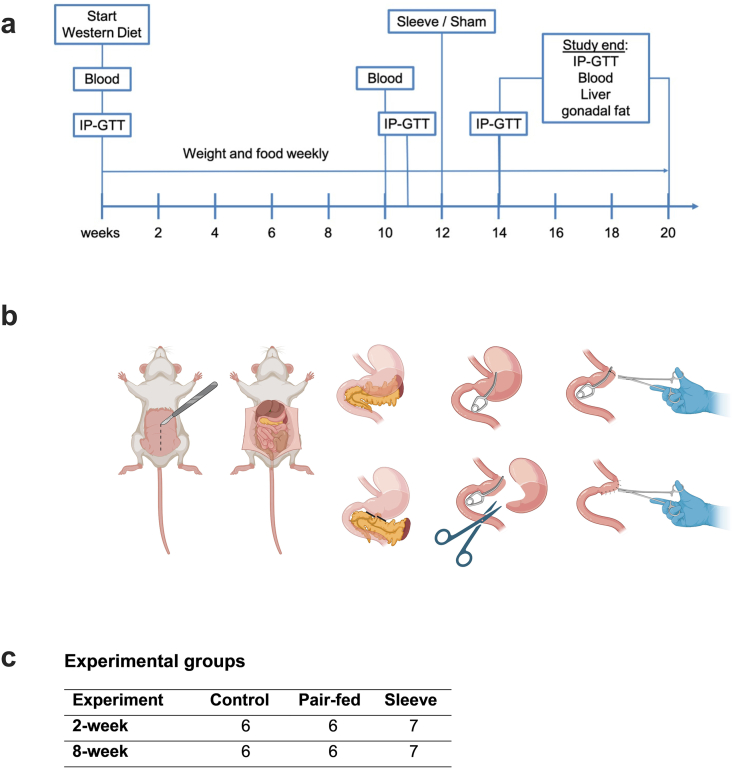
Study design and surgical procedure. (a) Mice were fed sucrose-enriched water (10 %) and a western diet with 40 kcal% fat, 20 kcal% fructose and 2 kcal% cholesterol (WD, D09100301, Research Diets, Inc., New Brunswick, NJ, USA) ad libitum at four weeks of age. After 12 weeks of diet mice were allocated to the experimental groups (control, Pf and SG) and underwent sham operation (control and Pf) or sleeve-gastrectomy. Food and weight were measured weekly. IP-GTTs and blood samples were collected at baseline, before surgery as well as 2 and 8 weeks after surgery. Two or eight weeks after surgery mice were sacrificed for organ explantation. (b) Representative pictures of the SG procedure. After midline laparotomy and mobilizing the stomach a non-traumatic surgical (vascular) clamp was placed on the resection margin. Then the stomach including the entire forestomach was resected and the remaining section of the stomach closed with a running suture (Created with BioRender.com). (c) Experimental groups.

### 2.3 Surgical procedure

Perioperative treatment was conducted as described before [21]. A midline laparotomy was performed to access the abdominal cavity, followed by the placement of an abdominal wall retractor. The stomach was then mobilized by carefully blunt-dissecting the loose connective tissue along the greater curvature. When required, the gastrosplenic ligaments and the pancreaticoduodenal arcade were cauterized and resected using an electrocautery device, while carefully preserving the pancreatic blood supply. The gastric sleeve was then created by placing a surgical clip reaching from the duodenum to the border between the forestomach and corpus without obstructing the esophagus. Then the stomach including the entire forestomach was resected and the sleeve finished with an 8-0 polypropylene running suture (Prolene®, Ethicon, Johnson & Johnson Medical GmbH, Norderstedt, Germany) (shown in Fig. 1b). The abdominal cavity was irrigated with saline, and the muscle layer was closed using a 6-0 polydioxanone suture (PDS®, Ethicon, Johnson & Johnson Medical GmbH, Norderstedt, Germany). The skin was sutured with interrupted stitches using 5-0 polypropylene suture (Prolene®, Ethicon, Johnson & Johnson Medical GmbH).

### 2.4 Sham procedure

In the sham operation, a laparotomy was performed, and the stomach was mobilized following the same procedure as in the SG. Afterwards, the surgical clamp was placed as described above, but without resection of the stomach. The clamp was removed, and the sham operation was completed following the same steps used for the SG.

### 2.5 Intraperitoneal glucose tolerance test (IP-GTT)

#### 2.5.1 Intraperitoneal glucose tolerance test (IP-GTT)

Mice were fasted overnight, and their fasting blood glucose levels were measured before administering an intraperitoneal glucose injection (2 g/kg of D-glucose). Blood glucose was then measured at 0 (fasting), 30, 90, 120, 150, and 180 min after glucose administration using whole venous blood collected from the tail vein (Accu-Chek Aviva, Roche Diabetes Care Deutschland GmbH, Germany). Glucose tolerance was compared by calculating the area under the glucose concentration-time curve.

### 2.6 Biochemical analysis

Blood samples were collected in heparin-coated tubes and centrifuged for analysis. Serum levels of alanine aminotransferase (ALT), cholesterol, and triglycerides were measured as markers of liver damage and metabolic function, following standard protocols at the Laboratory Facility of the Institute of Laboratory Animal Science, University Hospital RWTH Aachen.

### 2.7 Histopathological examination

Liver biopsies were taken, and the median lobe was fixed in 4 % paraformaldehyde. The liver tissue was then embedded in paraffin, and sections of 4 μm in thickness were prepared. To assess liver morphology and fibrosis, the sections were stained with hematoxylin and eosin (H&E), periodic acid-Schiff (PAS), and reticulin. A pathologist, blinded to the experimental groups, performed the histological evaluation and scoring. The Nonalcoholic Fatty Liver Disease Scoring System (NAS) was used to quantify steatosis (0–3), lobular inflammation (0–3), and hepatocellular ballooning (0–2) as described previously [22]. Fibrosis staging was carried out based on the criteria established by Kleiner et al. [23]. Immunohistochemistry was performed using monoclonal antibodies targeting F4/80^+^ macrophages (BM8, 14-4801-82, eBioscience™, Austria) and CD45^+^ leukocytes (14-0451-82, eBioscience™, Austria) were used. Photomicrographs were taken at 200× magnification and analyzed with the open-source software ImageJ.

### 2.8 Flow cytometry

Flow cytometry was carried out following previously established protocols [24].

### 2.9 Proteomics and downstream data analysis

For a detailed description of proteomic analysis and sample preparation, mass spectrometry and data analysis see supplementary document.

### 2.10 Statistics

The Shapiro-Wilk test was used to assess the normality of data distribution. Continuous variables are expressed as the mean ± standard deviation (SD). For data with a normal distribution, group differences were evaluated using one-way or two-way ANOVA, followed by Tukey's post hoc test for multiple comparisons. For non-normally distributed data, the Kruskal-Wallis test was applied along with Dunn's multiple comparison test. A p-value of less than 0.05 was considered statistically significant. All statistical analyses and graphical representations were performed using GraphPad Prism (version 7.0, La Jolla, USA).

## 3 Results

### 3.1 Body weight

Mice in all groups lost weight following SG or Sham operation and started to regain weight two weeks after surgery. In SG mice, % total body weight loss (%TWL) was significantly higher than that in Pf-sham and ad libitum-sham mice after 2 weeks and only than control mice after eight weeks (Fig. 2a–c).

**Fig. 2:**
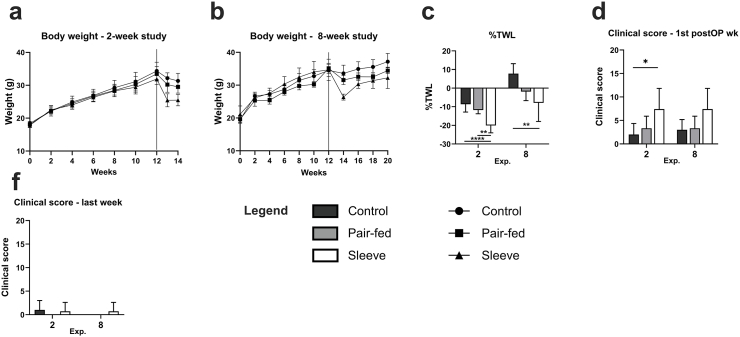
Body weight and clinical status. Monitoring of body weight throughout the (a) 2-week and (b) 8-week study. (c) %TWL before termination of the experiment was significantly higher in SG mice in the 2- and 8-week experiment. For animal welfare assessment a clinical scoring system was used. (d) shows the clinical score and degree of strain in the first postoperative week and (f) before termination of the experiment. ∗p < 0.05, ∗∗p < 0.01 and ∗∗∗p < 0.001.

Food intake did not differ between groups before surgery. Postoperative energy intake could not be measured during the first postoperative week due to the liquid diet and the stepwise introduction of food within the first week after surgery. In the eight weeks study, postoperative energy intake was significantly lower in Pf animals compared to SG or Sham groups. There was no difference between Sham and SG mice.

### 3.2 Clinical status

Clinical status was impaired in the first week after the operation with a higher score in the SG groups compared to the sham operation groups (Fig. 2 d and f). The maximum score during the entire experiment was 10 (DS2) a few days after surgery, but all mice recovered promptly after the operation with no further impact of animal welfare during the experiment.

### 3.3 Metabolic assessment

**Fig. 3:**
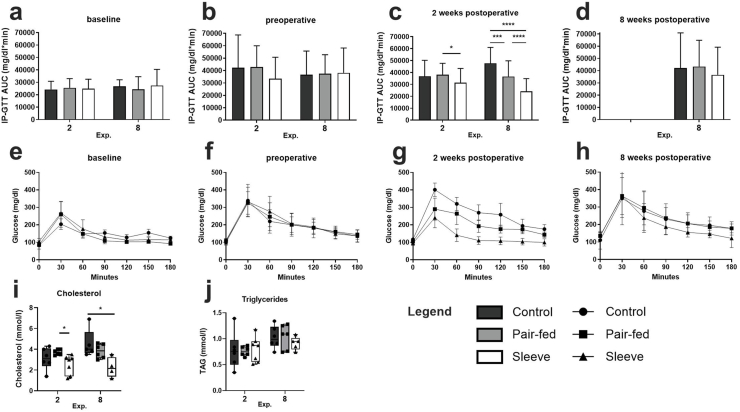
Metabolic assessments. (a, b, c, d) Area under the glucose time curve (AUC) of intraperitoneal glucose tolerance test (IP-GTT) for the 2- and 8-week experiments; (e, f, g, h) IP-GTT curves at baseline, preoperative, 2 weeks postoperative and 8 weeks postoperative of the 8-week study. (a–b) and (e–f) show no differences between the groups at the baseline and the preoperative timepoint. (c) Glucose tolerance improved after SG 2 weeks after surgery, which is depicted as a decrease in IP-GTT AUC compared to sham operated animals. (d) Regarding the long-term experiment, 8 weeks after surgery the IP-GTT AUC is still decreased after SG compared to control and Pf, but without being statistically significant. (i) cholesterol levels before termination of the experiment (2 and 8 weeks after surgery). Cholesterol levels are decreased in the SG groups 2- and 8-weeks following surgery. (j) Triglyceride levels do not differ between groups at both time points. ∗p < 0:05, ∗∗p < 0:01 and ∗∗∗p < 0:001.

To address the other components of the metabolic syndrome glucose tolerance, cholesterol and triglyceride levels were analyzed. As expected, baseline analysis and preoperative evaluation of glucose tolerance did not differ between groups in both experiments (Fig. 3a, b, e, f). Two weeks after surgery, however, glucose tolerance improved significantly in the SG groups compared to control and Pf animals in the long-term and to only Pf in the 2-week experiment (Fig. 3c and g). This effect deteriorated 8 weeks after surgery. The AUC of IP-GTT was still lower in SG animals, but without being statistically significant (Fig. 3d and h).

Cholesterol levels were lower after SG compared to both sham operation groups after two and 8 weeks, whereas the difference was only statistically significant compared to Pf in the 2-week and to the ad libitum control group in the 8-week experiment (Fig. 3i). In contrast to cholesterol, triglyceride levels did not differ between groups (Fig. 3j).

### 3.4 Liver injury

**Fig. 4:**
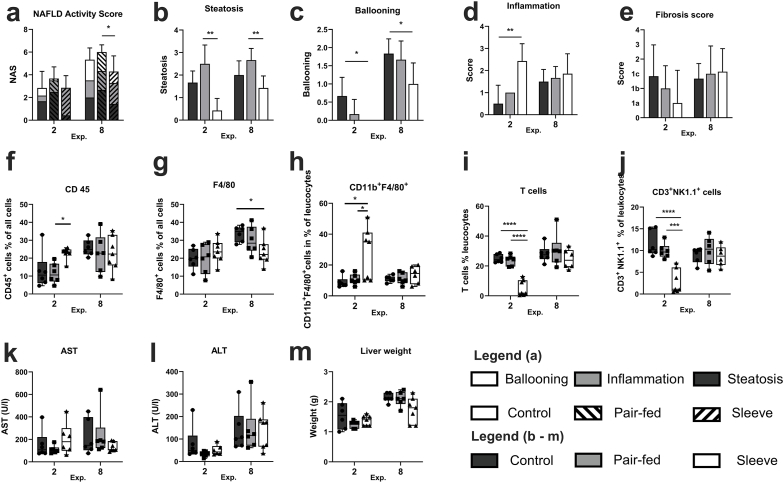
Liver injury. (a) NAFLD Activity Score (NAS) displayed the severity of MASH. (b–d) shows the different components (steatosis, inflammation, ballooning) of the NAS. (e) Fibrosis score according to Kleiner et al. (f) Immunohistological results of CD45^+^ and (g) F4/80^+^ cells, (h–j) Flow cytometry was used to further identify the immune cell types. (h) The proportion of CD11b^+^F4/80^+^ macrophages were significantly higher in SG mice compared to control and Pf mice 2 weeks after surgery. (i) T-cells were and (j) CD3^+^NK1.1^+^ cells were significantly decreased in SG mice compared to control and Pf mice 2 weeks after surgery. These effects were no longer detectable in the long-term study. (k) AST and (h) ALT levels in serum before termination of the studies. (m) Liver weight measured at explantation. Data represent mean ± SD; ∗p < 0:05, ∗∗p < 0:01 and ∗∗∗p < 0:001.

Nonalcoholic Fatty Liver Disease Activity Score (NAS) was determined to evaluate the effects of SG on MASH. NAS did not differ between groups in the 2-week experiment, whereas after 8 weeks the NAS of the SG group was significantly lower than of the Pf group, but again there was no significant difference between SG and the ad-libitum control group (Fig. 4a). However, a closer look at the different components of the NAS, presents a more distinguished pattern. In the 2-week experiment hepatic steatosis is decreased after SG with achieving statistical significance compared to Pf mice (Fig. 4b). Hepatocellular ballooning is also lower after SG compared to the control group (Fig. 4c). In contrast to ballooning and steatosis, hepatic inflammation is significantly increased in SG mice compared to control animals (Fig. 4d). These opposite manifestations of the different components result in the missing differences in the overall score but indicate major effects of SG on hepatic pathophysiology. In the long-term experiment we can still observe the decreased steatosis and ballooning after SG, but the effects on inflammation are diminished (Fig. 4b–d). In contrast to inflammation fibrosis scores did not differ between groups (Fig. 4e). To further evaluate hepatic inflammation immunohistology was performed. Interestingly, CD45^+^ leucocytes are increased two weeks after SG, whereas macrophages did not differ between groups. In contrast to the 2-week time point, 8 weeks after surgery macrophages are decreased in SG mice and CD45^+^ leucocytes did not differ between groups (Fig. 4f and g). Flow cytometry was used to further identify the immune cell types. In the 2-week experiment the proportion of CD11b^+^F4/80^+^ macrophages were significantly higher in the SG group compared to control and Pf animals (Fig. 4h). This difference was diminished 8 weeks after surgery. The inflammatory disturbances two weeks after SG were also shown by a significantly decreased proportion of T-cells and CD3^+^NK1.1^+^ cells in the SG group compared to both control and Pf groups (Fig. 4i and j).

To further evaluate hepatocellular injury, serum alanine aminotransferase (ALT) and aspartate aminotransferase (AST) levels were analyzed (Fig. 4k and l). Although ALT levels seem to increase during the postoperative time, levels of the transaminases did not differ between groups. Liver weight was decreased compared to control livers 8 weeks after SG, but without gaining statistical significance (Fig. 4m).

### 3.5 Liver proteome

**Fig. 5:**
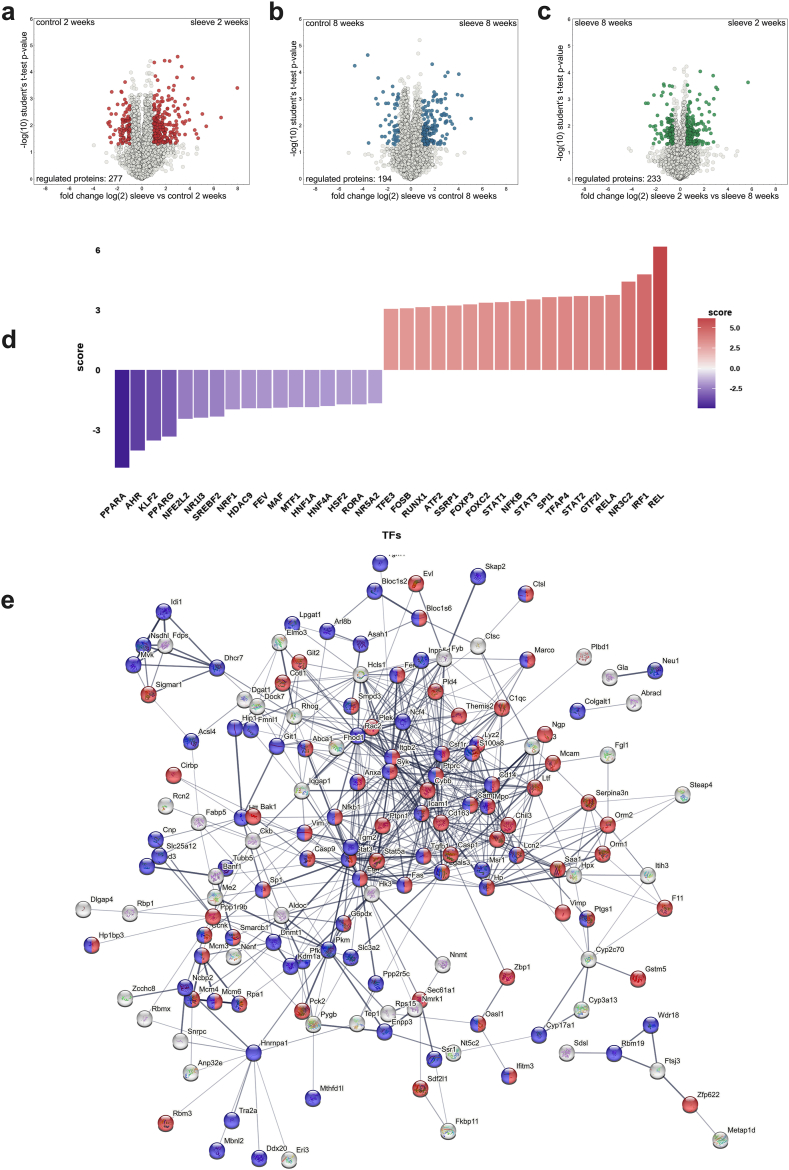
Proteomic analysis of the liver. (a–c) Volcano plots of proteins upregulated in (a) sleeve compared to control in the 2 weeks experiment, in (b) sleeve compared to control in the 8 weeks experiment and in (c) 2 weeks sleeves compared to 8 weeks sleeves. (d) Predicted regulation of transcription factor activities in the comparison of 2 weeks sleeve vs control using the decoupleR package (see supplementary text file for details). (e) PPI network of proteins upregulated in sleeve livers compared to control livers obtained from the 2-week experiment. Proteins involved in the stress/inflammation (including elevated neutrophil involvement, such as neutrophil infiltration)-associated pathways (using the (red color; GO0006950). Proteins associated with increased mortality or altered cellular aging patterns (blue color; MP 0010768 “Mortality/aging”). (a–b) minimum 2-fold regulation, p-value max. 0.05; (c) 1.5-fold level changes. PPI networks were generated using string-db.org. (For interpretation of the references to color in this figure legend, the reader is referred to the Web version of this article.)

In order to gather deeper insight in the organ response after surgery we conducted a proteomic analysis of liver samples from the individual groups described above. Around 4300 proteins were quantified after stringent filtering of the raw data (a protein had to be found at least in all samples of one of the groups in order to be included in the final list, see Table S1). Livers obtained from mice that underwent surgery appeared in a well separated cluster from the other samples (see Fig. S1a), in which the two time points are clearly separated as well. The comparison between the control situation and post-bariatric surgery after 2 weeks exhibited the most severe differences on the proteome level. A total of 277 proteins were found to be significantly regulated (minimum 2-fold regulation, p-value max. 0.05; see Fig. 5a). Analysis for enrichment of those proteins upregulated after bariatric surgery could be linked to stress/inflammation (including elevated neutrophil involvement, such as neutrophil infiltration)-associated pathways (using the STRING database; Fig. 5e; red color; GO0006950 “response to stress”, amongst others, see Table S2) as well as proteins associated with increased mortality or altered cellular aging patterns (blue color; MP 0010768 “Mortality/aging”). Proteins among these include such molecules as the Fas- and EGF-receptors, caspases 1 and 9, Stat3 and Stat5a as well as Haptoglobin (HP) and S100a8, which all show elevated levels in liver specimen two weeks after surgery. In contrast to this, less proteins were upregulated in the control situation (Fig. S1b), where only proteins involved in lipid biochemistry appear significant (red color). When investigating the potential modulation of transcription factor activities that might contribute to these protein differences, STAT1-3 and the NF-κB/Rel/RelA family appear upregulated in the liver two weeks after sleeve gastrectomy (Fig. 5d). In contrast, several well described liver modulators (PPARα, HNF1α and HNF4α) were found, amongst others, to exhibit reduced activity in the 2 weeks post SG livers. A similar, yet significantly less drastic situation was observed when comparing the control situation with post-bariatric surgery after 8 weeks on the proteomic level. Interestingly, less proteins (194 in total) were found to be significantly regulated (Fig. 5b) and the regulation was not as pronounced as in the 2-week comparison. Proteins with increased levels after 8 weeks post-bariatric surgery show again an enrichment of stress/inflammation (red) as well as altered mortality/aging associated processes (blue; see Fig. S2 and Table S2). While Egfr, Fas, Casp9, Hp and the Stat proteins were no longer found to be upregulated at 8 weeks after surgery, Casp1 and S100a8, amongst others, still exhibited elevated protein levels. In contrast, no relevant enrichments could be identified when analyzing proteins upregulated in the control situation after 8 weeks.

**Fig. 6:**
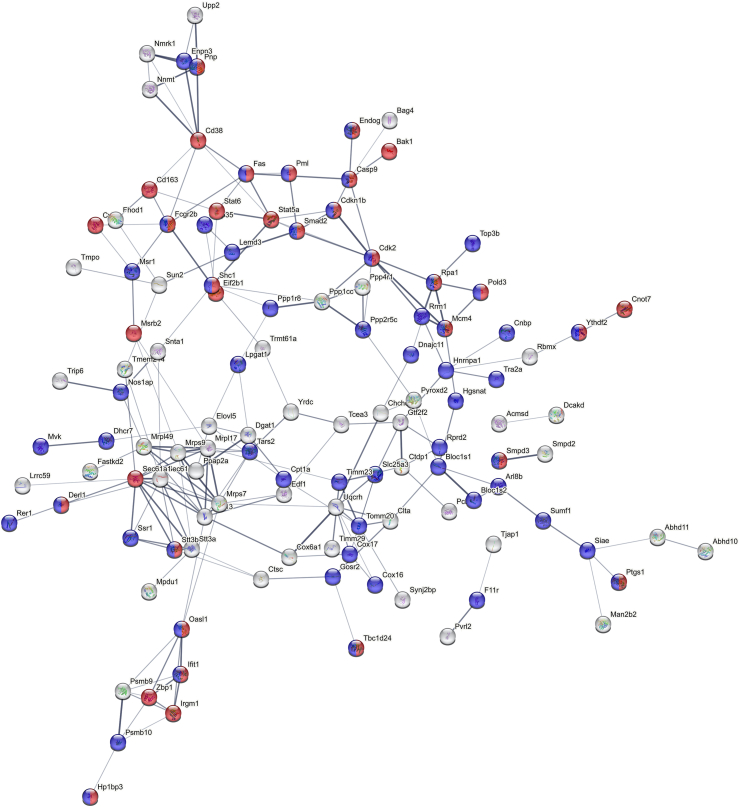
Proteomic analysis of the liver [2]. PPI network of proteins upregulated in sleeve livers of the 2-week experiment compared to sleeve livers of the 8-week experiment. Proteins involved in the stress/inflammation (including elevated neutrophil involvement, such as neutrophil infiltration)-associated pathways (using the (red color; GO0006950). Proteins associated with increased mortality or altered cellular aging patterns (blue color; MP 0010768 “Mortality/aging”). PPI networks were generated using string-db.org. (For interpretation of the references to color in this figure legend, the reader is referred to the Web version of this article.)

When comparing the individual sleeve surgery specimen, the analysis of enrichment provided a clear picture. Strikingly, when employing a more extended window of regulation (1.5-fold level changes; Fig. 5c, 233 regulated entries) proteins with enhanced levels 2 weeks post-surgery show a strong enrichment of the aforementioned stress/inflammation and mortality/aging-related ontologies (red and blue, respectively; Fig. 6), similar to the condition of control vs sleeve. Interestingly, these included Fas, Casp9, Smad2 as well as Stat5a and Stat6, suggesting increased levels of apoptosis shortly after surgical intervention. On the contrary, livers obtained from mice 8 weeks after surgery show an increase of many proteins involved in lipid metabolism. These findings support the conclusion that at this time a slow recovery from the initial high-stress/inflammation/neutrophil-involving phase is ongoing, which strongly corroborates the findings obtained from immunohistology (see above).

## 4 Discussion

Bariatric surgery is the most effective treatment modality for obesity and MASH with a vast amount of data proving its effects [15,25,26], but the mechanisms behind improved metabolism and liver pathophysiology are still under investigation [27]. Furthermore, there is a rare number of case series which report acute liver failure or impaired liver function following bariatric procedures [18,28].

We share these observations in our patient population. Whereas usually patient lose weight after bariatric procedures and liver function improves [29], we observed some patients with postoperative deterioration of liver function capacity using the LiMAx test [30]. Preoperative factors impeding liver function recovery included type 2 diabetes mellitus (T2DM), weight and male sex.

Due to important clinical implications like in these reports, we investigated the effects of SG on liver pathophysiology two and eight weeks after SG in a MASH mouse model. Body weight and liver steatosis decreased after SG compared to the control groups and glucose and cholesterol metabolism improved. These effects were more pronounced two weeks after surgery. Analysis of the livers two weeks after SG showed less liver steatosis and ballooning in SG mice, but significantly more inflammation. A closer look at the inflammatory infiltrate showed major differences between the groups. Within the immune cells the proportion of CD11b^+^F4/80^+^ macrophages were increased, whereas T-cells and CD3^+^NK1.1^+^ cells were decreased.

Natural killer (NK) and NKT cells are major player in liver homeostasis and hepatic inflammation and are involved in several hepatic diseases including MASH [31,32]. The role of NKT cells in liver pathophysiology is controversial in literature. Depending on the subtype it can be proinflammatory or even protective. Notably, bariatric surgery is able to affect the immune response. Moulin et al. showed that NK cell activity was reversed after Roux-en-Y gastric bypass (RYGB) [33]. In our study within the immune cells the proportion of CD3^+^NK1.1^+^ cells, were decreased. In another study of our group, a decreased proportion of CD3^+^NK1.1^+^ was associated with more severe MASH and toxic MASH diets [24]. Fortunately, the effects on the inflammatory infiltrate diminished 8 weeks after surgery.

According to the inflammatory infiltrate proteome analysis showed a massive stress response of the liver following the SG procedure. Proteins involved in apoptosis like Fas, Casp1 and Casp9 or in the acute phase response (Stat3, Haptoglobin) are upregulated in SG compared to control mice. This stress response genuinely decreases after 8 weeks, and lipid metabolic processes and lipid transportation is more pronounced 8 weeks after sleeve compared to the 2-week timepoint. A study investigating proteome profiling in human plasma after Roux-en-Y gastric bypass revealed similar dynamics of global inflammation status, drastically increasing after one week of surgery to gradually decrease throughout the investigation period, exhibiting already significantly lower inflammation levels after 12 weeks [34].

The proteomic analysis revealed several transcription factors that may contribute to the induction of a proinflammatory response (Fig. 5d). STAT1-3 and NF-κB/Rel/RelA, which play critical roles in inflammation and cell regulation [[35], [36], [37], [38]], are upregulated in the liver two weeks after sleeve gastrectomy. Interestingly, PPARα, a key transcription factor in hepatic lipid metabolism [39], and HNF4α, the master regulator of liver function [40], are both downregulated two weeks post-sleeve gastrectomy. These factors are essential for maintaining normal liver homeostasis. In the liver, HNF4α has been identified as a core transcription factor [41] and regulates genetic programs underlying the morphological and functional differentiation of hepatocytes [42]. In non-alcoholic steatohepatitis and high-fat diet-fed diabetic models, liver expression of HNF4-α is significantly reduced [43]. In rats with advanced cirrhosis, reduction in HNF4α expression correlates with worsening of liver function [44]. The combination of increased JAK/STAT and Rel signaling, along with reduced HNF4α signaling, may be key drivers of the inflammatory disturbances following sleeve gastrectomy.

However, the question of whether changes in inflammation-related signals and gene expression are the cause or consequence of hepatic improvement following surgery remains under debate. Recent data suggests that metabolic improvements precede the resolution of inflammation after bariatric surgery [45].

To fully understand the effects of bariatric surgery on hepatic pathophysiology and inflammation, it is essential to consider weight loss-independent effects on gastrointestinal hormone and bile acid homeostasis, particularly those involving the farnesoid X receptor [46] and its targets [47]. Additionally, a recent report on the gastrointestinal peptides proguanylin (GUCA2A) and prouroguanylin (GUCA2B), which play major roles in obesity and satiety, demonstrated a protective effect against lipotoxicity and mitochondrial dysfunction. These peptides might contribute to improved MASLD outcomes following bariatric surgery [48]. Thus, gastrointestinal hormones and signaling pathways should be a key focus of future research on MASLD and bariatric procedures.

Taken together, we observed major inflammatory and regulatory disturbances in the liver two weeks after SG. There are several explanations for these disturbances. Firstly, the rapid and drastic weight loss causes the release of free fatty acids from the adipose tissue, which increases fatty acid delivery to the liver, leading to hepatic lipotoxicity. Other mechanisms which are more likely to follow malabsorptive bariatric procedures are protein malnutrition, bacterial overgrowth in the blind loop and gut microbiota alterations [18,19,49,50].

However, these inflammatory disturbances were no longer detectable or decreased in the long-term study. The rapid weight loss and decrease of hepatic fat after SG lead to a proinflammatory response in the liver, which shifts to a more moderate immune response in the long-term study. We suggest that the liver experiences major injury in the early phase after SG following the surgical trauma and the excess of free fatty acids and concomitant oxidative stress. Under healthy circumstances, the liver exhibits a sufficient regenerative capacity to cope with this massive stress response and is able to improve histopathological findings and liver function in the long-term. However, pre-injured livers or livers with concomitant liver diseases might not overcome this stress response and respond with acute liver failure or progressive fibrosis. Therefore, it is of most urgent importance to identify patients who are at risk for acute or chronic liver failure.

In our patients we calculate the NAFLD fibrosis score (NFS) of every patient preoperatively. Patients with a pathological score are referred to a hepatologist preoperatively. If there are no concomitant liver diseases patients are scheduled for bariatric surgery. In all patients with a macroscopically affected liver a liver biopsy is taken intraoperatively for histopathological evaluation and calculation of NAS and SAF scores. Patients with a NAS of 1–2 are observed in the standard post bariatric follow-up. Patients with an intermediate score of 3–4 are referred to a hepatologist for an ultrasound of the liver and fibroscan after weight loss (usually 6 months after surgery). Patients with a score of ≥5 are dismissed with an appointment for immediate hepatological counseling.

## 5 Conclusions

SG demonstrates significant benefits in treating obesity and MASH. Nonetheless, the liver's stress response in the early phase after surgery warrants careful consideration, especially in patients with pre-existing liver conditions. Continued research is needed to better understand the mechanisms of improved liver function after bariatric surgery and to develop tailored approaches to optimize patient outcomes and mitigate potential risks to the liver.

## Ethics statement

All animal experiments were approved by the governmental care and use committee (LANUV), Recklinghausen, NRW, Germany, granted official permission (84–02.04.2014.A356) and conducted in accordance with the federal German law and European directive 2010/63/EU on the protection of animals used for scientific procedures. Our experiments were also in compliance with the ARRIVE guidelines and the Guide for the Care and Use of Laboratory Animals (8th edition, NIH publication, 2011, USA).

## Funding

This work was supported by the 10.13039/100010345B. Braun Foundation, Melsungen, Germany (BBST-S-16-00030).

## Institutional review board statement

The animal study protocol was approved by the governmental care and use committee (LANUV), Recklinghausen, NRW, Germany, granted official permission (84–02.04.2014.A356) and conducted in accordance with the federal German law and European directive 2010/63/EU on the protection of animals used for scientific procedures.

## Data availability statement

The mass spectrometry proteomics data have been deposited to the ProteomeXchange Consortium (http://proteomecentral.proteomexchange.org) via the PRIDE partner repository [51] with the dataset identifier PXD044185; Reviewer account details: Username: reviewer_pxd044185@ebi.ac.uk; Password: nWBMv2xB.

## CRediT authorship contribution statement

**Andreas Kroh:** Writing – review & editing, Writing – original draft, Visualization, Methodology, Investigation, Funding acquisition, Formal analysis, Data curation, Conceptualization. **Sophia Schmitz:** Writing – review & editing, Formal analysis, Data curation. **Saskia Köhne:** Investigation. **Julia Andruszkow:** Investigation, Formal analysis. **Jochen Nolting:** Methodology. **Christian Preisinger:** Writing – original draft, Visualization, Methodology. **Karsten Große:** Investigation. **Roman M. Eickhoff:** Visualization, Investigation, Data curation. **Daniel Heise:** Investigation. **Thorsten Cramer:** Supervision, Resources. **Karl Peter Rheinwalt:** Methodology. **Patrick Hamid Alizai:** Investigation. **Ulf Peter Neumann:** Supervision, Resources. **Tom Florian Ulmer:** Supervision, Conceptualization.

## Declaration of competing interest

The authors declare the following financial interests/personal relationships which may be considered as potential competing interests: Andreas Kroh reports financial support was provided by B. Braun Foundation, Melsungen, Germany. All other authors declare that they have no known competing financial interests or personal relationships that could have appeared to influence the work reported in this paper.

## Figures and Tables

**figs1:**
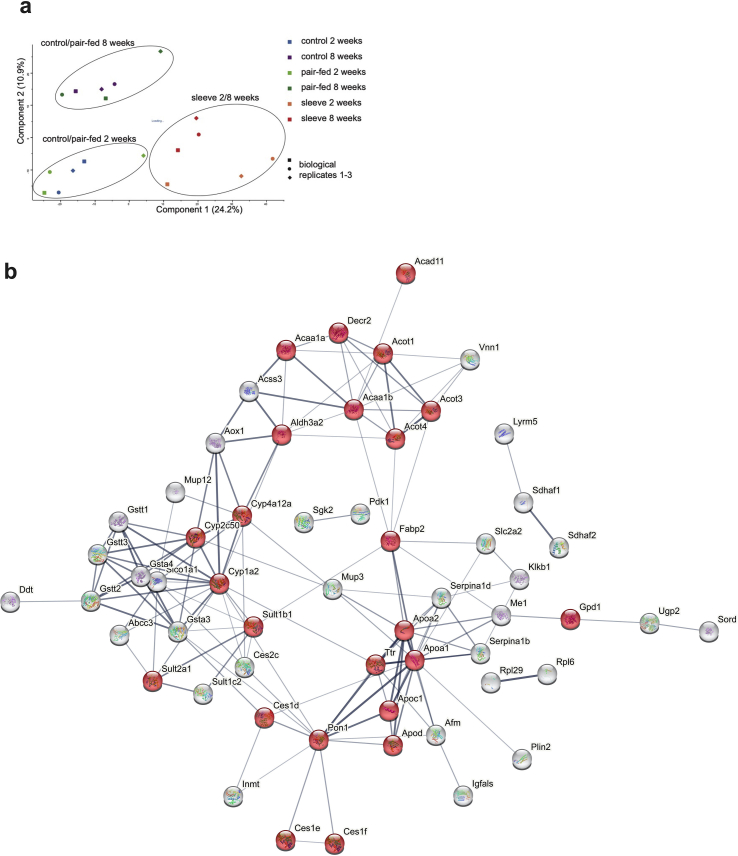
Figure S1 Proteomic analysis of the liver (a) Principal component analysis plot indicates clustering of the individual biological replicates from liver tissue samples obtained from mice that underwent sleeve-gastrectomy. The two time points are separated as well. (b) PPI network of proteins upregulated in control livers compared to sleeve livers of the 2-week experiment. Proteins involved in lipid biochemistry appear significant (red color). Minimum 2-fold regulation, p-value max. 0.05

**figs2:**
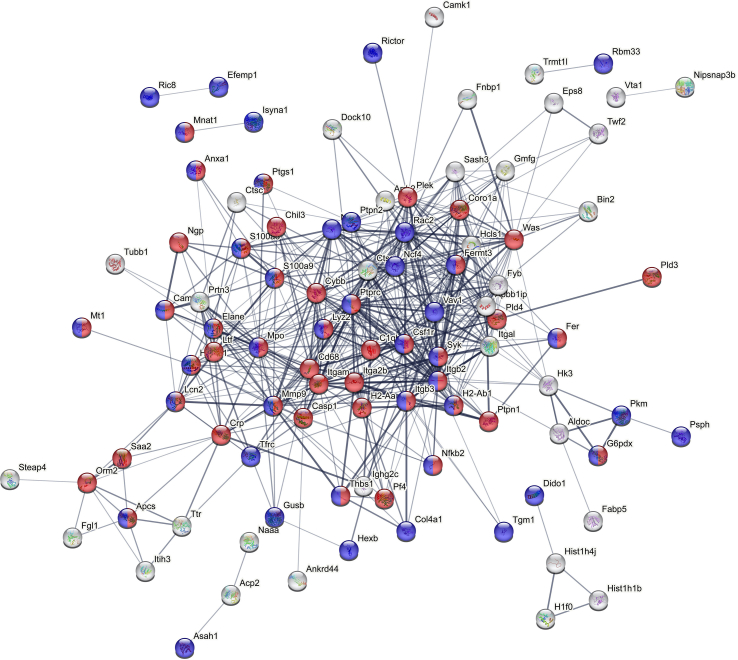
Figure S2. Proteomic analysis of the liver (2) PPI network of proteins upregulated in sleeve livers compared to control livers obtained from the 8-week experiment. Proteins involved in stress/inflammation (red) as well as altered mortality/aging associated processes appear significant. Minimum 2-fold regulation, p-value max. 0.05
