# Supplementary material for: Sleeve-gastrectomy results in improved metabolism and a massive stress response of the liver proteome in a mouse model of metabolic dysfunction-associated steatohepatitis
Source: Heliyon. 2024 Sep 28;10(21):e38678. doi: 10.1016/j.heliyon.2024.e38678 (PMC11550656; doi:10.1016/j.heliyon.2024.e38678)
Supplement: Multimedia component 1 [file mmc1.docx]

**Materials and Methods**

**Proteomics and downstream data analysis**

Mouse livers were excised and snap frozen in liquid nitrogen. Six livers were used per condition, where two livers were combined for each analysis (n=3 for each condition). All samples were lysed in ice cold urea buffer (8M urea in 50 mM ammoniumbicarbonate (ABC)) for 30 minutes. The resulting protein lysates were then cleared by centrifugation for 15 min at 4°C and 21000g in a bench-top centrifuge. The protein concentration of the individual supernatants were determined by using a bicinchoninic acid assay (Thermo Scientific). Each lysate (1 mg protein) was reduced by treatment with 4mM dithiothreitol (DTT) in 50 mM ABC for 30 min at 56°C; subsequently the samples were alkylated using 10 mM iodoacetamide in 50 mM ABC for 30 min in the dark at room temperature. The samples were then digested with LysC (1:100 w/w, Wako) in 8 M urea for 4 hours at 37°C. The reaction was then diluted with 3 volumes of 50 mM ABC to yield a 2 M urea solution. Trypsin was then added (1:100 w/w, Thermo Scientific) and the mixture was left for digestion overnight at 37°C. On the next day the samples were acidified with formic acid (FA) to yield a final concentration of 3% FA, cleaned up with Sep-Pak C18 1 cc Vac Cartridges (Waters), lyophilized in a speed-vac and stored at -80°C. Sample preparation for mass spectrometer (MS) analysis was carried out by fractionation of 100µg of each sample using a high pH reversed-phase fractionation kit (Thermo Scientific, cat.no. 84868) according to the manufacturer’s instructions. Samples were concatenated to result in five fractions (fractions 1+8, 2,3, 4+5, 6+7). All fractions were dried down and then resuspended in 3% FA/5% acetonitrile (ACN) and loaded onto a nanoLC system (RSLCnano, Thermo Scientific). For MS analysis, an approach using a trapping (Acclaim PepMap100, C18, 5 µm, 100 Å, 300 µm i.d. × 5 mm, Thermo Scientific) and an analytical column (Easyspray 50 cm column (ES803; Thermo Scientific) was used. Settings were as follows: peptide separation on the analytical column was carried out at 45°C with a 150 min gradient: Peptide separation was carried out using a 165 minute gradient 0–10 min: 5% buffer B (buffer A: 0.1 % FA; buffer B: 80 % acetonitrile, 0.1 % FA), 10–104 min: 5–35 % buffer B, 104-114 min: 35-45 % buffer B, 114-114.1 min: 45-95 % buffer B, 114.1-119 min: 95% buffer B, 119-120 min: 95-5 % buffer B, 120-150 min: 5 % buffer B. MS analysis was done on a Q Exactive plus mass spectrometer (Thermo Scientific) in data dependent mode with a spray voltage of 2kV and the capillary temperature set to 250°C. MS settings: 70,000 resolution; AGC target: 1e6; maximum injection time: 50 milliseconds; scan range: 350-1600 m/z. dd-MS2 settings were: 17,500 resolution; AGC target: 1e5; maximum injection time: 55 milliseconds; top 20 precursor fragmentation; isolation window: 2.0 m/z; normalized collision energy: 27. dd settings were: minimum AGC: 2e3; 30 seconds dynamic exclusion; only 2^+^ to 5^+^ peptides.

The resulting raw data was analysed using the MaxQuant software suite (version 1.6.17.0) with the built-in Andromeda search engine ([1](#_ENREF_1)). The *Mus musculus* UniProt database version 12/2020 (only reviewed and canonical sequences) was searched using MaxQuant default settings with Trypsin as the protease (two missed cleavages allowed). Carbamidomethylation on cysteine residues was set as the fixed modification whereas both methionine oxidation and N-terminal protein acetylation were set as variable modifications. The false discovery rate (FDR): was set at 0.01 on both the protein and PSM levels; the minimum peptide length was seven amino acids. Relative quantification of the proteins was done using the label free quantification algorithm from MaxQuant.

The resulting proteinGroups.txt file from the MaxQuant search was imported into Perseus (version 1.6.14.0, ([2](#_ENREF_2))). Grouping was done for all the biological replicates of the individual groups (six groups; sleeve surgery 2 and 8 weeks, pair-fed 2 and 8 weeks, and control 2 and 8 weeks). Entries representing reverse hits, contaminants, and “only identified by site” were removed; furthermore, all proteins with less than two unique peptides were discarded from the list. Two-sample tests were performed on the six different groups using the default settings from Perseus. These analyses were then used for generation of the Volcano plots (using Instant Clue ([3](#_ENREF_3))). The settings for the volcano plot were p-value ≤ 0.05 and a ratio of ≥ 2-fold change (>1.30103 (–log (10) p-value) and >2 (log (2) ratio). Protein ratios fulfilling these requirements were considered significantly regulated.

STRING (Search Tool for the Retrieval of Interacting Genes/Proteins) analysis for protein clustering and GO term enrichments was performed using STRING v11 ([4](#_ENREF_4)) at string-db.org.

Analysis of potentially involved transcription factors was carried out with the protein list from the comparison sleeve 2 weeks vs control 2 weeks using decoupleR as described on github: (<https://saezlab.github.io/decoupleR/articles/tf_bk.html>) (([5](#_ENREF_5)); version 2.9.7)

**References**

1. Tyanova S, Temu T, Cox J. The MaxQuant computational platform for mass spectrometry-based shotgun proteomics. Nat Protoc 2016;11:2301-2319.

2. Tyanova S, Temu T, Sinitcyn P, Carlson A, Hein MY, Geiger T, Mann M, Cox J. The Perseus computational platform for comprehensive analysis of (prote)omics data. Nat Methods 2016;13:731-740.

3. Nolte H, MacVicar TD, Tellkamp F, Kruger M. Instant Clue: A Software Suite for Interactive Data Visualization and Analysis. Sci Rep 2018;8:12648.

4. Szklarczyk D, Gable AL, Lyon D, Junge A, Wyder S, Huerta-Cepas J, Simonovic M, et al. STRING v11: protein-protein association networks with increased coverage, supporting functional discovery in genome-wide experimental datasets. Nucleic Acids Res 2019;47:D607-D613.

5. Badia IMP, Velez Santiago J, Braunger J, Geiss C, Dimitrov D, Muller-Dott S, Taus P, et al. decoupleR: ensemble of computational methods to infer biological activities from omics data. Bioinform Adv 2022;2:vbac016.
